# Using isolation-by-distance to jointly estimate effective population density and dispersal distance: a practical evaluation using bumble bees

**DOI:** 10.1007/s00442-025-05721-4

**Published:** 2025-05-29

**Authors:** Dylan T. Simpson

**Affiliations:** 1 Grad Program in Ecology, Rutgers University, New Brunswick, NJ USA; 2 Negaunee Institute for Plant Science, Conservation, and Action, Chicago Botanic Garden, Glencoe, IL US; 3 Plant Biology and Conservation, Northwestern University, Evanston, IL US

**Keywords:** Relatedness, *N*_e_, Effective population size, Neighborhood size, Movement

## Abstract

Effective population density and intergenerational dispersal distance are key aspects of population biology, but obtaining empirical estimates of these parameters can be difficult. This is especially true for my study taxa, wild bees. In this paper, I apply and evaluate an existing but underutilized method to estimate effective density and dispersal distance of bumble bees (*Bombus*, Apidae). Specifically, using 10 datasets of bumble bees in North America, I use the relationship between genetic isolation-by-distance and Wright’s neighborhood size to define a density-dispersal isocline—that is, a curve describing pairs of density and dispersal values consistent with observed rates of isolation-by-distance. These parameters are inversely related; as one increases the other decreases. I then use outside estimates of bumble bee dispersal distances to make more specific estimates of effective colony density. Compared to some prior estimates of census density (100s to 1000s colonies/km^2^), my estimated effective colony densities were very low (1–41 effective colonies/km^2^). I also hypothesize, however, that these estimates are affected by the spatial extent of sampling, due to scale-dependent patterns in the distribution of individuals. To test this hypothesis, I subsampled each dataset to simulate varying study extent, and repeated my analysis. Within populations, effective densities tended to decrease when measured across larger spatial extents. Altogether, I demonstrate a useful and under-appreciated tool for studying population biology, especially of small, mobile animals like bees, but also show that researchers must interpret their results carefully within the context of their study design.

**Supplementary Information:**

The online version contains supplementary material available at 10.1007/s00442-025-05721-4.

## Introduction

Bees are a diverse group of insects that provide crucial pollination services to crops and wild plants (Klein et al. 2007; Ollerton 2017; Reilly et al. 2020; Simpson et al. 2022). While bees have received much attention in recent years due to concern over pollinator declines, much of this attention has focused on community-level patterns in abundance or diversity. Meanwhile, surprisingly little attention has been paid to population-level processes of most bee species (Dorian et al. 2024). Here, I focus on two important but understudied aspects of bee population biology: effective population size and intergenerational dispersal.

Effective population sizes and intergenerational dispersal are key aspects of any species’ population biology (Wright 1969; Allendorf et al. 2013). Effective population size (hereafter, *N*_e_) is a genetic measure of population size related to the strength of genetic drift. Intergenerational dispersal is the distance between where an individual is born and where it reproduces (sometimes called ‘parent–offspring distance’). Together, these demographic parameters affect the rate of gene flow, the efficiency of selection and the potential for local adaptation (Lenormand 2002; Kawecki and Ebert 2004; Bachmann et al. 2020). From a conservation perspective, *N*_e_ and dispersal rates are critical because they also affect population viability by determining rates of inbreeding and demographic rescue (Hanski 2011). Despite their importance, however, little is known about *N*_e_ or dispersal distances of bees, likely because they are difficult to study.

Like for any small, highly mobile animal, measuring bee dispersal distance is extremely challenging. Directly observing bee dispersal would require following a bee from where it emerges to where it builds its own nest, and doing so over any meaningful distance would be next to impossible. A more feasible method for estimating dispersal distances, at least for eusocial bees, is genetic mark-recapture (Lepais et al. 2010; Carvell et al. 2017; Mola and Williams 2019): locations of workers are recorded in one year and the location of their sister queens is recorded the following year, and the distance between these is used to estimate of how far the queen dispersed from her natal nest. This method is promising for providing a robust lower bound on dispersal distances (Mola and Williams 2019). However, this method is also very labor intensive and is likely to underestimate long-distance dispersal events because longer-distance dispersal is more likely to go beyond the extent of the study region where it cannot be observed.

Estimating *N*_e_ is especially difficult for bees because bees are haplodiploids. Typically, *N*_e_ is measured using genetic samples from individuals in a population. Of the methods to do so, only the ‘sibship’ method has been adapted for use with haplodiploids (Wang 2016). The sibship method has been successfully applied to bees (e.g. López-Uribe et al. 2015; Vickruck and Richards 2017), but it has two shortcomings. First, it measures the immediate *N*_e_ of the previous generation (Wang 2009) and thus does not account for variability in population size over time. As a result, estimates may be inconsistent between years and not representative of a population’s genetic diversity and long-term rates of genetic drift. Second, for eusocial bees, this method is only meaningful if queens are sampled, because variability in worker number among colonies is unrelated to effective population size (Crozier 1979). Yet queen bees are active for shorter periods of time and in lower abundances than worker bees, making studies based on queens more challenging.

**Fig. 1:**
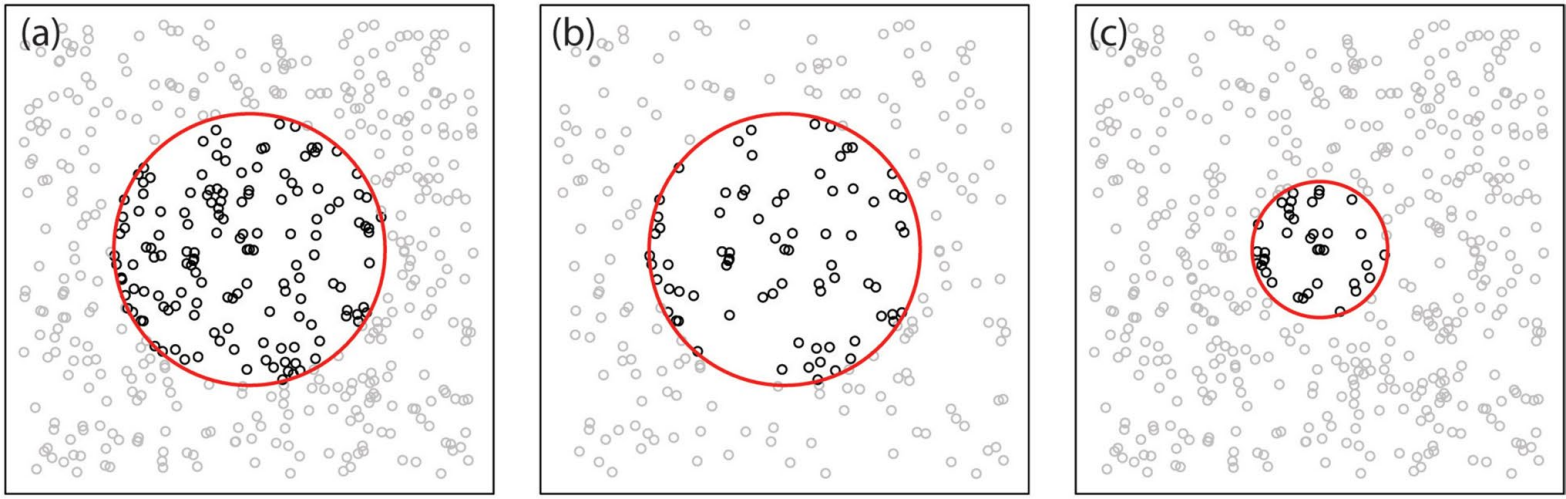
Illustration of the genetic neighborhood and neighborhood size. Each panel shows the position of individuals in a population and the red circles highlight the genetic neighborhood for an individual in the center. The neighborhood is the area within a distance of 2*σ*, where *σ* is the standard deviation of dispersal distance. The neighborhood thus contains the population the focal individual is most likely to encounter after dispersing. Neighborhood *size* is the number of individuals in the neighborhood (in the red circle) and is determined by population density (compare panels **a** and **b**) and dispersal distance, as measured by *σ* (compare **a** and **c**)

In this study, I demonstrate a promising way around these limitations: to use the rate of genetic isolation-by-distance (IBD) to jointly estimate effective population density (i.e., *N*_e_ per area) and dispersal distance. Isolation-by-distance is the tendency for individuals or (sub)populations that are farther apart to also be more distantly related, or ‘genetically distant.’ The strength of IBD—that is, the rate at which genetic distance increases with geographic distance—depends on that population’s “neighborhood size,” which represents the size of the local breeding pool (or local *N*_e_; Fig. 1). Neighborhood size is itself a function of effective population density and dispersal (Wright 1943). Larger neighborhood sizes (those with greater density and/or dispersal) mean weaker IBD, and vice versa. This relationship is such that neighborhood size can be estimated directly by regressing genetic distance against geographic distance (Rousset 1997; Watts et al. 2007; Robledo-Arnuncio and Rousset 2010; see Methods). Finally, this neighborhood size estimate can be decomposed into joint estimates of mean effective population density and dispersal distance—that is, paired estimates of density and dispersal that are compatible with observed IBD. If one of these is known, the other can be directly estimated (e.g. Pinsky et al. 2010, 2017; Antoni and Saillant 2017). Otherwise, observed rates of IBD can be used to define a density-dispersal isocline—a curve of possibilities consistent with the data (Dupont et al. 2015; François et al. 2021). While this isocline does not provide a single definitive estimate for *N*_e_ or dispersal, it can help define reasonable bounds for both.

This IBD-based method should be a useful complement to existing methods in wildlife ecology (for bees or any other taxa). Particularly useful for bees, it can easily account for haplodiploidy (Rousset 1999). It is also compatible with how surveys are conducted for many landscape or population genetics studies and it describes spatiotemporal averages in density and dispersal (Robledo-Arnuncio and Rousset 2010), which could provide a useful check on the local, short-term estimates provided by the sibship and genetic mark-recapture methods (e.g. Lepais et al. 2010; López-Uribe et al. 2015; Carvell et al. 2017; Vickruck & Richards 2017). Yet, while this method has seen some use in other taxa, it has not yet been used for bees (but for a similar approach see Zayed & Packer 2001).

There are, however, two potential issues with the IBD method that have not been previously explored. Both problems relate to the spatial scale of sampling. The first is that having too large of a study extent may downwardly bias IBD estimates and upwardly bias neighborhood size estimates. This is partly because the IBD relationship is not perfectly linear (Rousset 1997), and partly because populations may not be in mutation-drift equilibrium across long distances (Slatkin 1993; Hardy and Vekemans 1999; Benestan et al. 2021). For both these reasons, IBD estimates measured across too great a distance are expected to be biased low, and IBD that might be apparent at smaller extents could appear weaker or disappear at larger extents (Benestan et al. 2021). Such an effect would upwardly bias estimates of neighborhood size.

The second reason estimated IBD might depend on the extent of observation is that IBD results from the *spatial average* of effective density and dispersal (Robledo-Arnuncio and Rousset 2010). As a result, IBD estimates could be affected by spatial patterns in the distribution of individuals and populations. In particular, I predict one could get different answers if sampling at a broad spatial extent, across which a species is more patchily distributed, versus sampling the same species at a smaller extent, where it is distributed more continuously (I illustrate this in Supplemental Information S1). A patchier distribution means more unoccupied space, which should result in a lower average density and thus stronger IBD. This is not to say that spatial heterogeneity creates bias—these average estimates are valid and spatial heterogeneity in density is certainly relevant to a species’ population biology. In estimating IBD and neighborhood size, however, such spatial heterogeneity will act as a confounding variable unless it is understood and accounted for. For each of these reasons, it is important to understand how observed IBD changes with the spatial scale of sampling before drawing any conclusions about the focal population.

In this paper, I use IBD to derive joint estimates of effective population density and dispersal distance for six North American bumble bee species. In doing so, I also evaluate the method and its sensitivity to spatial scale. My analysis uses a large dataset of *Bombus impatiens* workers I collected in southern New Jersey, USA, as well as nine publicly available datasets that were previously published by others (Table S2.1; Lozier et al. 2011; Jha and Kremen 2013; Jha 2015; Jackson et al. 2018). For the population represented by each dataset, I ask (1) What is Wright’s neighborhood size? (2) What are corresponding estimates of effective population density and dispersal distance? And (3) How do estimates these estimates change with the spatial scale of sampling?

## Materials and methods

### Study species

In this study I focus on six North American bumble bee species: *Bombus bimaculatus*, *B. impatiens*, *B. occidentalis*, *B. pensylvanicus*, *B. vancouverensis* and *B. vosnesenskii*. Like all non-parasitic bumble bees, these are eusocial species with annual colonies (Williams et al. 2014). New queens and males are produced at the end of the season, after which mating occurs, males and old queens die, and mated queens overwinter to establish the next year’s colonies. Thus, dispersal by males would necessarily occur in the fall, while dispersal by queens could occur in the fall and/or following spring.

What is known about *N*_e_ or dispersal of bumble bees? Like for bees generally, very little. The best dispersal estimates of which I am aware come from Europe, using the genetic mark-recapture method. In one study, Carvell et al. (2017) report mean dispersal distances by *B. terrestris*, *B. pascuorum* and *B. lapidarius* as 1500 m, 1150 m, and 980 m, respectively. In another study, Lepais et al. (2010) report individual estimates of dispersal distance by 177 *B. lapidarius* queens. Distances were right skewed with a mean of 2 km. Given the limitations of the genetic mark-recapture method, however, these are likely underestimates.

I am not aware of any reliable estimates of bumble bee effective population sizes. Some studies have reported *N*_e_ estimates (e.g. Geib et al. 2015; Mola et al. 2020), but these were calculated assuming ideal populations. Actual *N*_e_ is typically far less than that of an ideal population, but exactly how much less varies by population and taxon (Frankham 1995).

### Methods for New Jersey Bombus

I genotyped 978 *B. impatiens* workers from 100 sites in southern New Jersey (Fig. S2.1), which I collected across two years. In 2020, I collected 486 bees from 79 sites by net or vane trap and, in 2021, I collected 492 bees from 21 different sites by net. Altogether, sites were spread across southern New Jersey’s dominant landcover regimes—urban/developed, crop agriculture, mixed and deciduous forest, and pine barrens. For those collected by net, sampling was always done as close to a single point as feasible, and always within 100-m of the center location. In all, pairwise distances among sites ranged from < 1 km to 146 km (mean = 40, sd = 22). After capture, specimens were either pinned and dried (2020 specimens) or placed in 95% ethanol and frozen at -30°C (2021 specimens).

I genotyped each specimen at 11 microsatellite loci, following established methods. Briefly, I extracted DNA from the two mid legs using Ampure beads (Ali et al. 2016), then used a multiplex PCR adapted from previous studies (Jha 2015; McGrady 2018; McGrady et al. 2021). Fragment analysis was performed by the New Jersey Medical School Genomics Center, and alleles were scored using Thermo Fisher Connect. Further details can be found in Supplementary Information S3.

After genotyping, I used the package genepop (Rousset 2008) in R (R Core Team 2024) to check each locus for deviation from Hardy–Weinberg equilibrium and the presence of null alleles, and pairs of loci for linkage disequilibrium. None of these tests were significant. Finally, I used the program COLONY (Jones and Wang 2010) to determine siblingship among workers, assuming monandrous mating (Supplementary Information S3). Among the 978 genotyped workers, I detected 843 unique colonies across 98 sites. In my IBD analyses, I used one randomly chosen worker per colony (thus *n* = 843 bees, 98 locations).

### Other datasets

I also analyzed nine publicly available datasets in which the original authors measured isolation-by-distance within a bumble bee population (Table S2.1). These were not meant to be a comprehensive assessment of North American *Bombus*, but instead as a set of case studies. These include four datasets on *B. vosnesenskii* at different spatial scales (Lozier et al. 2011; Jha and Kremen 2013; Jha 2015; Jackson et al. 2018) and one dataset each for *B. vancouverensis* (Jackson et al. 2018), *B. impatiens*, *B. bimaculatus*, *B. pensylvanicus*, and *B. occidentalis* (Lozier et al. 2011). These datasets include individual genotypes and locations of *Bombus* workers from unique colonies, which are the only required data for this analysis. Genotypes were based on microsatellites or SNPs (single nucleotide polymorphism), and bees were collected across dozens to thousands of kilometers. Note: the dataset on *B. vancouverensis* was published as describing *B. bifarius*, which was later split into *B. bifarius *sensu stricto and *B. vancouverensis* (Ghisbain et al. 2020). The dataset I used here is firmly within the range for *B. vancouverensis* and so I describe it as such.

### Analysis

The theoretical basis of my analysis is the relationship between genetic distance and geographic distance, which is mediated by effective density and dispersal. In two dimensional space, $genetic\, distance\propto \frac{\text{ln}\left(geographic\, distance\right)}{neighborhood\, size}$, where genetic distance is measured as *F*_st_/(1 − *F*_st_), *â*, or *ê* (Rousset 1997, 2000; Watts et al. 2007). Following Rousset (1999) and assuming equal sex ratios, neighborhood size for bees is ${N}_{\text{b}}= 3{D}_{\text{e}}\pi {\sigma }^{2}$, where *D*_e_ is effective population density (i.e. *N*_e_ per unit area), $\pi$ is the mathematical constant, and *σ* is the standard deviation of axial dispersal distance (Rousset 1997), which is more easily thought of as the standard deviation of the dispersal kernel (Pinsky et al. 2010). Equal sex ratios should be a safe assumption because, while bumble bee colonies tend to produce more males than queens (Müller and Schmid-Hempel 1992), bumble bee queens rarely mate with more than one male (Payne et al. 2003; Bird et al. 2024). Thus the “extra” males should contribute little to effective population size.

To estimate effective neighborhood sizes of each *Bombus* species (Q1), I regressed genetic distance among samples against the natural logarithm of geographic distance (Rousset 1997, 2000). Neighborhood size is estimated as the inverse of the slope of this regression (i.e. ${\widehat{N}}_{\text{b}}=1/\beta$). For the previously published datasets, I measured genetic distance between pairs of sites as *F*_st_/(1 − *F*_st_) (Rousset 1997). This metric is intended to compare discrete populations, but is also recommended when individuals are sampled in groups from discrete sites (Watts et al. 2007; Robledo-Arnuncio and Rousset 2010). For my dataset, I measured genetic distance using Rousset’s *â*, which measures pairwise genetic distance between individuals. I used this metric because many of my sampling locations included too few specimens to calculate *F*_st_. I used Rousset’s *â* instead of Watts’ *ê* because the higher precision of *ê* comes at the cost of bias and I preferred not to bias the estimate for only one of 11 datasets. I performed these analyses with the package genepop for R (Rousset 2008; R Core Team 2024), which reports 95% ‘approximate bootstrap confidence’ intervals in IBD slope. In four datasets, confidence intervals for the slope of IBD crossed 0 to include negative values. Realistically, IBD must be ≥ 0; IBD = 0 is the null expectation of a panmictic population, while having IBD < 0 would mean further individuals are *more* related. Thus, in cases where confidence intervals of the IBD slope crossed 0, I consider the slope to have a lower confidence bound of 0. Because ${\widehat{N}}_{\text{b}}$ is the inverse of IBD and ${0}^{-1}=\infty$, ${\widehat{N}}_{\text{b}}$ in these cases is considered to have an upper confidence bound of infinity. This is simply to say that, in these four datasets, there was only enough information to define a lower bound for neighborhood size, not an upper bound.

To generate joint estimates of effective density and dispersal (Q2), I first defined a range of potential values for *D*_e_ and *σ*. Then, for a series of values across that range for one parameter, I used the neighborhood size estimates (${\widehat{N}}_{\text{b}}$) from Q1 to solve for the corresponding value of the other parameter. With ${N}_{\text{b}}= 3{D}_{e}\pi {\sigma }^{2}$, density and dispersal can be estimated as ${\widehat{D}}_{\text{e}}=\frac{{\widehat{N}}_{\text{b}}}{3\pi {\sigma }^{2}}$ and $\widehat{\sigma }=\surd \left(\frac{{\widehat{N}}_{b}}{3{D}_{\text{e}}\pi }\right)$. I report confidence intervals by solving for *D*_e_ and *σ* using the upper and lower CIs of the neighborhood size estimate. I then visualize results as curves of *D*_e_ against *σ*, which I refer to as joint-estimate isoclines. Without further information, however, it cannot be known where along these isoclines these population actually are. So, to narrow in on a realistic values for *D*_e_ and *σ*, I used estimates of dispersal by *B. pascuorum* from Lepais et al. (2010) to estimate *σ* (see Supplementary Information S4), and found $\widehat{\sigma }$ = 2.3. Given the limitations of mark recapture estimates, however, this $\widehat{\sigma }$ should be interpreted as something of a lower bound on *σ*.

To test the effect of spatial scale on parameter estimates (Q3), I subset each dataset to simulate sampling across a series of spatial extents. I then recalculated IBD using those subsets. To better ensure IBD could be estimated within subsets, I used only the six datasets in which significant IBD was detected in the full dataset. To be sure that results were driven by spatial extent and not changes in sample size, I randomly sampled site-pairs to maintain constant sample size across scales. Then, because this result will also be driven by this stochastic sampling process, I repeated this process 1000 times for each scale in each dataset and report the median and distribution of point estimates for each scale. To determine which scales I could test with each dataset, I found with the 50th percentile of pairwise distances between sites (i.e., the pairwise distance that would include 50% of observations), then tested a series of 20 scales between the 50th percentile distance and the maximum (including the maximum but excluding the 50th percentile itself, because there could be no variation among subsamples). This means that the scales tested for each dataset differ, but that each tested scale uses 50% of its respective dataset.

To report the effects of scale, I describe trends in the median estimate across the 1000 subsamples. To make the results more interpretable, I use my outside estimate of $\widehat{\sigma }$ = 2.3 to report *D*_e_ (like elsewhere in Results). It is important to note, however, that the shape of the *D*_e_ ~ scale relationship does not depend on *σ*, only on *σ* itself being independent of scale, which I think is a safe assumption. To quantitatively describe trends in *D*_e_ estimated across scales, I also fit general additive models (GAMs) to the median *D*_*e*_ estimate using the gam function in the mgcv package in R (Wood 2003; R Core Team 2024). These models predicted median *D*_e_ with a single smoothing term for scale (i.e., ${D}_{\text{e}}\sim s(scale)$). To avoid overfitting in cases where median estimates were more erratic, I limited the dimensions of the smoother to *k* = 5.

### Assumptions

While stated elsewhere where they are immediately relevant, I restate my analytical assumptions here for clarity and transparency: (1) To calculate neighborhood size for bees, I assume equal sex ratios. Because they are haplodiploid, a male-biased population would decrease neighborhood size relative to the formula I used. I believe assuming equal sex ratios is safe, though, because queens typically mate with only one or very few males; extra males will thus contribute little. (2) Relatedly, to determine siblingship among workers in my dataset, and to convert from effective individuals to effective colonies, I assumed monandrous mating. Queens in the subgenus *Pyrobombus* (which includes the species *B. bimaculatus*, *B. impatiens, B. vancouverensis*, and *B. vosnesenskii*) are known to occasionally mate with more than one male. In determining siblingship, though, statistically estimating mating number (i.e., males per queen) typically leads to greater bias than assuming monandry (Bird et al. 2024). In converting to effective colony number, assuming mating numbers > 1 would lead to slightly lower effective colony numbers. (3) The IBD method assumes that populations are in equilibrium between mutation, migration and genetic drift. This is likely not the case at large spatial scales, which is part of the motivation for assessing the effects of scale. (4) To report specific point estimates of effective density, I assume the dispersal parameter *σ* = 2.3, which I estimates using an independent dataset on *B. pascuorum*, a European bumble bee species (Lepais et al. 2010). It is unknown how much dispersal varies among species and so this estimate of *σ* should be taken as a starting point only. (5) In describing the effect of spatial scale on effective density estimates, I assume that the dispersal parameter *σ* is constant across scales. I have no reason to expect that dispersal rates are dependent on spatial scale in the same way density might be. If it is though, this assumption should not qualitatively affect the result.

## Results

**Fig. 2:**
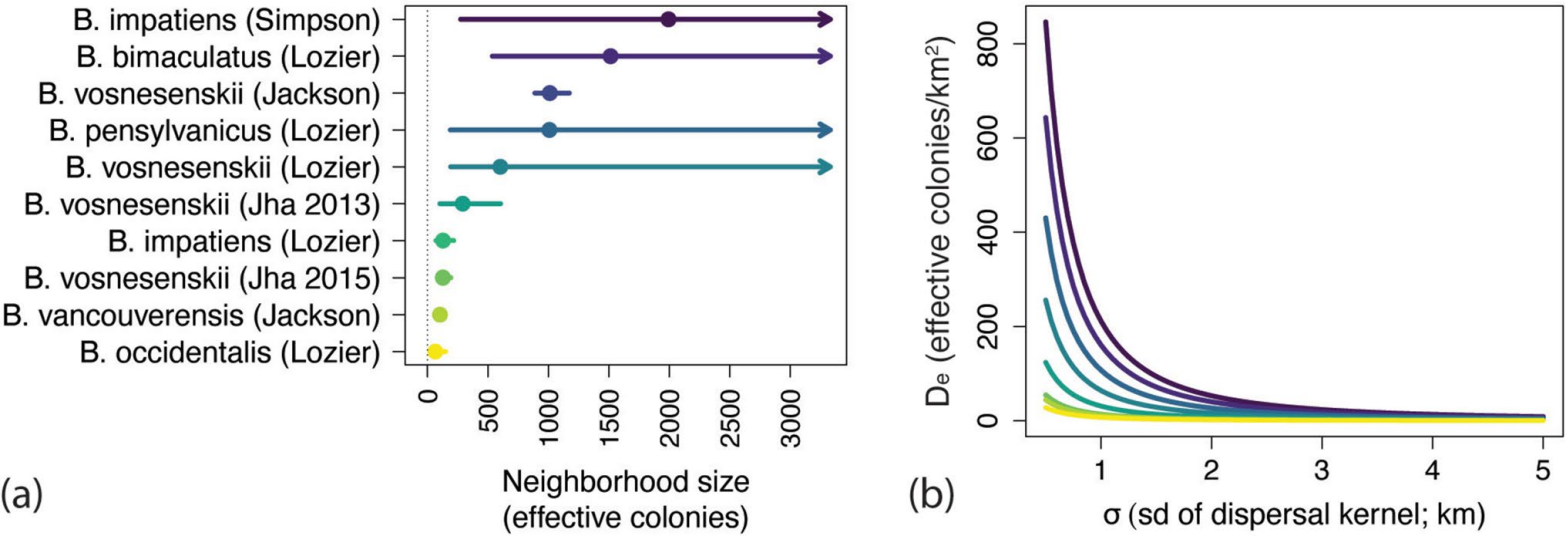
Neighborhood size estimates and 95% CIs (**a**), decomposed into joint effective density (*D*_e_) and dispersal distance (*σ)* estimates (**b**) for each dataset. The name in parentheses denotes the original publishing author. The curves in **b** are isoclines that describe pairs of effective colony density and dispersal consistent with the neighborhood size estimates in **a**. The arrows in **a** denote CIs that go to infinity

Across datasets, point estimates of neighborhood size (${\widehat{N}}_{\text{b}}$) ranged from 98 to 2991 effective individuals. Assuming monandrous mating, this corresponds to 65 to 1994 effective colonies (Fig. 2a) (as haploids, males contribute less to *N*_e_ than queens, and so effective colonies = 2/3 × effective individuals; Crozier 1979). Smaller neighborhood estimates tended to be more precise. This is because ${\widehat{N}}_{\text{b}}$ is calculated as the inverse of the IBD slope estimate and so ${\widehat{N}}_{\text{b}}$ increases rapidly as the slope estimate approaches 0. As a result, between two similarly precise IBD estimates, the estimate closer to 0 will produce a larger confidence interval for ${\widehat{N}}_{\text{b}}$.

For most datasets, realistic values of bumble bee dispersal correspond to low effective densities (Fig. 1b). Using my dispersal distance estimate for *B. pascuorum* of $\widehat{\sigma }$ = 2.3 km, effective population densities ranged from ${\widehat{D}}_{\text{e}}$ = 1.3–41 (mean = 14; CIs = 0.68 to infinity) effective colonies/km^2^. In those datasets in which upper confidence bounds could be estimated, CIs ranged from ${\widehat{D}}_{\text{e}}$ = 0.68–24 effective colonies/km^2^, suggesting the higher point estimates in other populations are likely overestimates. And, given that $\widehat{\sigma }$ = 2.3 km is likely an underestimate of σ, effective densities could be even lower: by *σ* = 5 km, ${\widehat{D}}_{\text{e}}$ ranges from 0.28 to 8.5 effective colonies/km^2^.

**Fig. 3:**
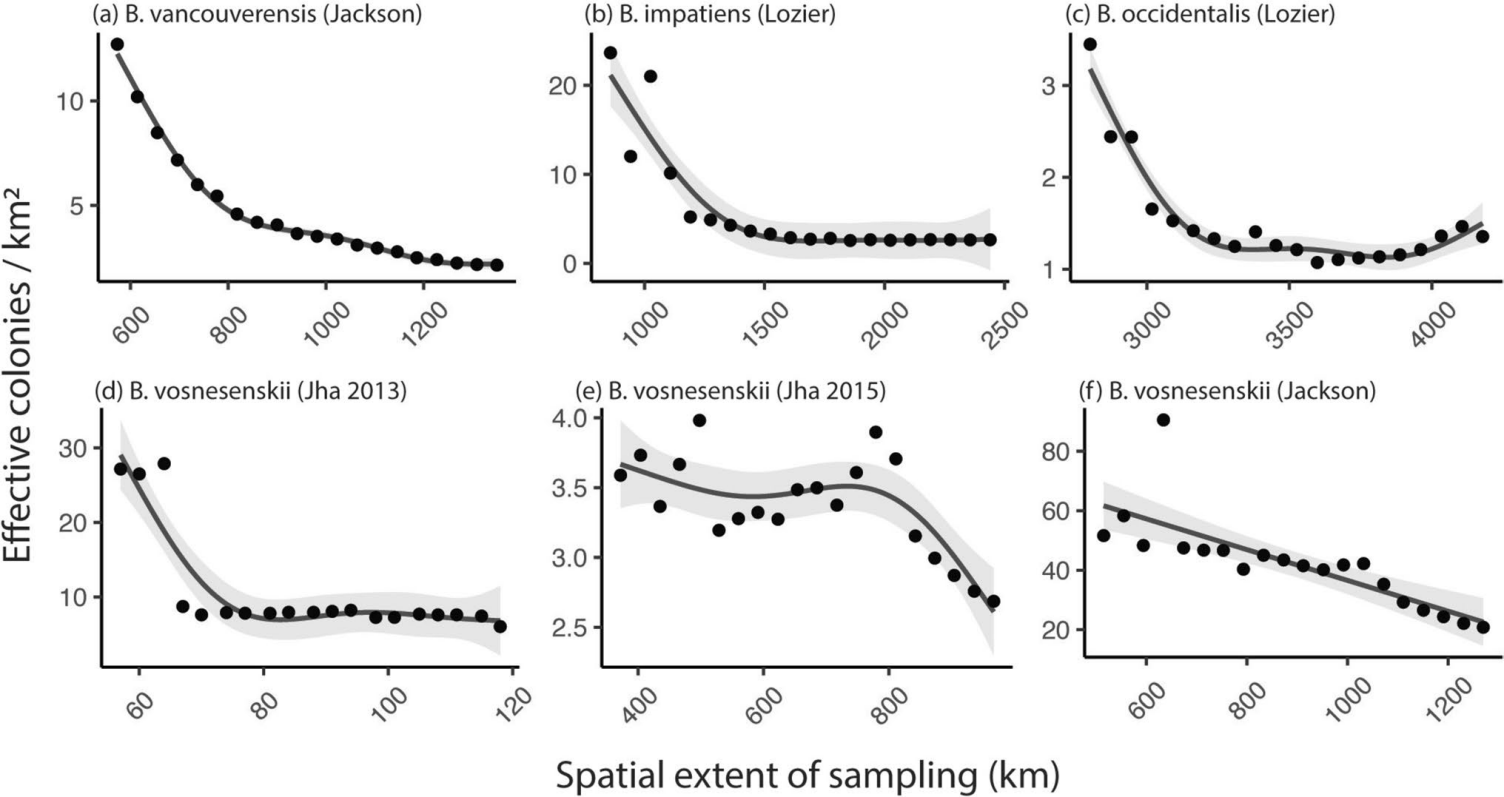
Observed rates isolation-by-distance—and thus effective density and dispersal estimates—change with the spatial extent of study. For illustration, I show effective density estimates, assuming constant dispersal across scales. In each panel, points are the median effective colony density estimate from 1000 subsampled site pairs, where estimates were made using pairwise site comparisons at less than or equal to the distance given on the horizontal axis and assuming the dispersal parameter *σ* = 2.3. Lines and ribbons are from represent expected values and 95% confidence intervals from general additive models. For each tested extent, 50% of the total observed pairwise site comparisons were used, so that the sample size underlying each density estimate is the same

Spatial extent affected IBD estimates in every dataset, though the effect was more pronounced in some than in others (Figs. 3, S5.1). In general, the observed IBD relationships were stronger at larger extents (i.e. when measured over longer distances). Assuming constant *σ*, this means effective density estimates would be lower at larger extents. The GAMs describing *D*_*e*_ as a function of scale were all highly significant (*p* ≤ 0.001; Fig. 3; Table S5.1). For *B. vancouverensis*, ${\widehat{D}}_{\text{e}}$ decreased consistently across all scales, from ca. 600–1400 km. Estimates for B*. impatiens* decreased rapidly from ca. 850–1200 km, then decreased more slowly before leveling off at ca. 1700 km. Estimates for *B. occidentalis* follow a similar pattern but at a larger scale, decreasing from ca. 2800–3600 km before leveling off or maybe increasing. In *B. vosnesenskii*, estimates drop sharply between ca. 50 and 70 km, then remain fairly stable until ca. 700 km before continuing to decline.

## Discussion

Despite their ecological importance, estimates of intergenerational dispersal distance and effective population size of bees have remained elusive. In this study, I used observed rates of genetic isolation-by-distance to derive joint estimate isoclines of effective population density (*D*_e_) and dispersal distances (*σ*) of six North American bumble bee species. By themselves, these isoclines are useful because they narrow the potential parameter space for *D*_e_ and *σ* estimates, but they are especially useful when combined with independent estimates of either *D*_e_ or *σ.* Using published estimates of dispersal by *Bombus pascuorum* queens (Lepais et al. 2010), I estimated *σ* to be least 2.3 km (see Supplementary Information S4). If dispersal is similar in the focal species of this study, my results suggest effective population densities of ca. 1.3–41 effective colonies/km^2^. Among the datasets for which I could fully estimate confidence intervals, the upper bounds of density for were all ≤ 24 effective colonies/km^2^. To my knowledge, these are the first robust estimates of bumble bee effective colony densities.

These effective colony density estimates are much lower than many—but not all—census density estimates for bumble bees. To some extent, this is to be expected. Effective population size is almost always lower than census population size, usually by about an order of magnitude (Frankham 1995). In some cases, census density estimates are in line with this expectation, ranging from ca. 10–100 colonies/km^2^ (Darvill et al. 2004; Knight et al. 2005; Wood et al. 2015). In other cases, though, estimates range from hundreds to thousands of colonies/km^2^ (Harder 1986; O’Connor et al. 2012, 2017; Iles et al. 2019; Pugesek and Crone 2021). These are greater than my estimates by up to three orders of magnitude. One reason for these differences among estimates could be differences in study species and location. Another reason could be spatial scale.

Estimates of population density likely depend on the spatial scale at which they are made. In the case of the census density estimates I described above, the highest estimates are those made in a small area of seemingly good habitat (Harder 1986; O’Connor et al. 2012, 2017; Iles et al. 2019; Pugesek and Crone 2021). In contrast, the smaller estimates were made by averaging across the foraging ranges of their focal populations (Darvill et al. 2004; Knight et al. 2005; Wood et al. 2015). These estimates might better represent the population as a whole because it takes into account landscape heterogeneity. Similarly, in the case of my effective density estimates, it is important to remember that they represent averages broad spatial scales. Such broad study areas are more likely to be heterogenous with respect to habitat quality and population density, such that the average density at this broader scale is likely lower than local density in good habitat. Thus, in heterogeneous landscapes, estimates of *D*_e_ should be smaller when IBD is measured at larger extents. Indeed, this is evidenced by my analysis of sensitivity to spatial scale.

In most datasets, IBD estimates increased, and average neighborhood size estimates decreased, with increasing spatial extent. Broadly, this suggests that many of the neighborhood size—and thus effective density—estimates I report in Results are much smaller than might be found locally. For example, assuming σ = 2.3, ${\widehat{D}}_{\text{e}}$ for *B. vancouverensis* increased from 2.1 to 13 effective colonies/km^2^ when study extent decreased from 1350 to 573 km (data from: Jackson et al. 2018). Similarly, ${\widehat{D}}_{\text{e}}$ for *B. impatiens* increased from 2.7 to 24 effective colonies/km^2^ when study extent decreased from 2440 to 858 km (data from: Lozier et al. 2011). This pattern is also consistent with a comparison to the *B. impatiens* I collected in New Jersey—in these specimens, IBD was measured across only 146 km and average ${\widehat{D}}_{\text{e}}$ = 41 effective colonies/km^2^ (though this estimate was very imprecise).

This effect of scale was non-linear and varied in shape among populations. In *B. vancouverensis*, ${\widehat{D}}_{\text{e}}$ consistently decreased with increasing scale, but in a decelerating fashion. Similarly, ${\widehat{D}}_{\text{e}}$ for *B. impatiens* and *B. occidentalis* decreased to a point and then leveled off (or maybe even increased slightly in *B. occidentalis*). *Bombus vosnesenskii*, which was represented here by three datasets, was more variable. At small scales, (60–120 km), *B. vosnesenskii* looks much like the other species, with a sharp drop to a flat line (Fig. 2d). At intermediate scales, ${\widehat{D}}_{\text{e}}$ is more erratic, appearing flat from 400 to 700 km, then declining (Fig. 2e). At larger scales, ${\widehat{D}}_{\text{e}}$ declines steadily and linearly from 600 to 1200 km (Fig. 2f). I hypothesize this variation in the effects of scale—that is, changes in the slope of the ${\widehat{D}}_{\text{e}}$ ~ scale relationship across species and populations—is due to variation in how “patchy” populations appear at different scales (sensu lacunarity; Plotnick et al. 1996; Supplementary Information S1). Under this hypothesis, scales across which ${\widehat{D}}_{\text{e}}$ changes are scales at which patchiness changes; scales across which ${\widehat{D}}_{\text{e}}$ remains constant are scales across which populations are similarly patchy. Testing this hypothesis, however, is beyond the scope of this study.

There is also a theoretical expectation that the strength of isolation-by-distance will become weaker at large spatial extents. As a result, neighborhood size (and thus ${\widehat{D}}_{\text{e}}$) estimates should become biased-high when estimated across large distances. This is because of the slightly non-linear relationship between genetic and geographic distance and because, across large distances, populations are less likely to be in equilibrium between drift, mutation, and gene flow (Slatkin 1993; Rousset 1997; Hardy and Vekemans 1999; Benestan et al. 2021). This expectation is not immediately apparent in my results, except *perhaps* for *B. occidentalis* (Fig. 2c). This could mean that these biases are not present in these data, or that they are counteracted by the opposing effect of spatial extent, as described above. Regardless, this theoretical expectation for bias at large extents suggests that estimates of neighborhood size—and thus effective density and dispersal—will be most reliable when estimated across smaller extents.

The assumption that populations be in equilibrium between drift, mutation, and geneflow also means that IBD relationships can be disrupted by things like recent range expansions. Thus, in addition to be being more reliable at smaller extents, the IBD-regression method should be most consistent when used within a species’ historic range.

Given these caveats and contingencies, how should these results or this method be used? Ideally, in parallel with focused study of census population density and environmental heterogeneity. Considering effective population size, there is a lot to be gained by being able to make direct comparisons of effective to census population size in the same population. This will advance our baseline understanding of bee population ecology and provide a point of comparison for at-risk species. For at-risk species, like *B. occidentalis* or *B. affinis*, including estimates of effective population density will help better understand those populations’ vulnerability to extirpation. Notably, a low effective colony density does not necessarily mean these populations are only few nests away from extirpation, but could mean they are more vulnerable to negative feedbacks that afflict small populations (Zayed and Packer 2005; Zayed 2009). Considering dispersal distances, IBD provides a method to assess rates of *effective*—i.e., successful—dispersal. This too will advance our baseline understanding of bee ecology, but will be especially useful for habitat management for at-risk species. An important part of habitat management is habitat and resource connectivity, which requires an animal’s successful dispersal through a landscape. Better understanding dispersal distances can thus improve management of habitat for connectivity.

Like any analysis, the precision of the IBD-regression method can depend on data quantity and quality. Here, this was most evident in comparing the results between datasets using microsatellites vs. SNPs. The two datasets that used SNPs (Jackson et al. 2018) yielded much more precise estimates of isolation-by-distance than the microsatellite datasets. This difference is because of the number of loci sequenced. These SNP datasets were produced using RADseq (Baird et al. 2008), which relies on next-generation sequencing and produces genotypes for hundreds to thousands of SNP loci, as compared to the microsatellite datasets, which in these cases include genotypes for 7–12 microsatellite loci. While one microsatellite locus carries more information than one SNP locus, the sheer volume of SNPs attained from RADseq gives them greater resolving power (Hauser et al. 2011; Gärke et al. 2012). In practice, however, this difference between methods will matter most for populations with large neighborhood sizes and weak IBD (i.e., slopes near 0).

Regardless of the molecular techniques, the IBD-regression method I demonstrate here is a useful tool for estimating effective population density and intergenerational dispersal. This is especially true for species in which dispersal is difficult to estimate in the field, or for haplodiploids, like bees, for which conventional methods for estimating effective population size are invalid (Wang 2016). Even in the absence of any estimates of dispersal or effective density, the joint estimate isoclines produced by this method constrain the possibilities to pairs of parameter values and can thus be used to infer reasonable bounds for both based on natural history knowledge. Any additional information, such as the independent estimates of dispersal I used here, can be used to further narrow in on realistic estimates.

Another attractive aspect of this method is that it is relatively inexpensive and easy to apply. While there are alternative methods that may be able to better parse dispersal and effective density (Ringbauer et al. 2017), these alternatives rely on large amounts of genomic data. Acquiring such datasets is expensive, especially for a large number of samples, and requires more laboratory and analytical expertise. The method I describe here can be applied with microsatellites, which are very inexpensive by comparison, and require relatively basic laboratory and analytical skills.

Due to these advantages, the IBD-regression method should be especially useful to field ecologists who are not experts in molecular ecology but are trying to better understand the population biology of their focal taxa. Particularly for bees or other insects, which can be especially difficult to study, this method provides a way to narrow in on estimates for dispersal and effective density that can otherwise be so difficult to measure.

## Supplementary Information

Below is the link to the electronic supplementary material.

Supplementary file1 (PDF 1146 KB)

## Data Availability

All data to recreate the analyses and figures are available at: 10.5061/dryad.ngf1vhj55. This includes original data collected for this study and re-formatted copies of data by other authors, which I included so my analyses could be recreated. Any use of these previously published data, however, need to cite the original authors.

## Code availability

All code used to run the analyses and create the figures in this paper are available in the same Dryad repository: 10.5061/dryad.ngf1vhj55.
