# Supplementary material for: Using isolation-by-distance to jointly estimate effective population density and dispersal distance: a practical evaluation using bumble bees
Source: Oecologia. 2025 May 29;207(6):93. doi: 10.1007/s00442-025-05721-4 (PMC12122646; doi:10.1007/s00442-025-05721-4)
Supplement: Supplementary file 1 — Supplementary file1 (PDF 1146 KB) [file 442_2025_5721_MOESM1_ESM.pdf]

Supplementary Information

Using isolation-by-distance to jointly estimate effective population density and dispersal distance: a practical evaluation using bumble bees. Dylan T Simpson. Oecologia, 2025.

Contents

S1: Why the spatial scale of study might affect estimates of IBD and effective density 2

Figure S1.1 2

Figure S1.2 3

Figure S1.3 4

S2: Datasets 5

Table S2.1 5

Figure S2.1 6

S3: Molecular analyses of New Jersey *Bombus impatiens* 7

S4: Outside estimates of dispersal 8

Figure S4.1 9

S5: Supplemental Results 10

Table S5.1 10

Figure S5.1 10

References 11

S1 Why the spatial scale of study might affect estimates of IBD and effective density

S1.1 Conceptual argument

Patterns in the spatial distribution of populations can depend on the spatial extent of observation – that is, the size of the landscape observed and sampled (Levin 1992, Plotnick et al. 1996). As an example, consider a population whose individuals are aggregated (i.e., patchily distributed) at a regional scale (Fig. S1.1, middle). Within a smaller landscape – as would be observed by a study performed across a smaller extent – this population can easily appear continuous (Fig. S1.1, top left). Even if the sampled landscape is not fully occupied, the occupied area is more likely to be continuously populated (Fig. S1.1, bottom left). At larger extents, it is still possible to observe a continuous population (Fig. S1.1, bottom right), but it is more likely that a larger focal landscape will include a discontinuous, or patchy population (Fig. S1.1, top right).

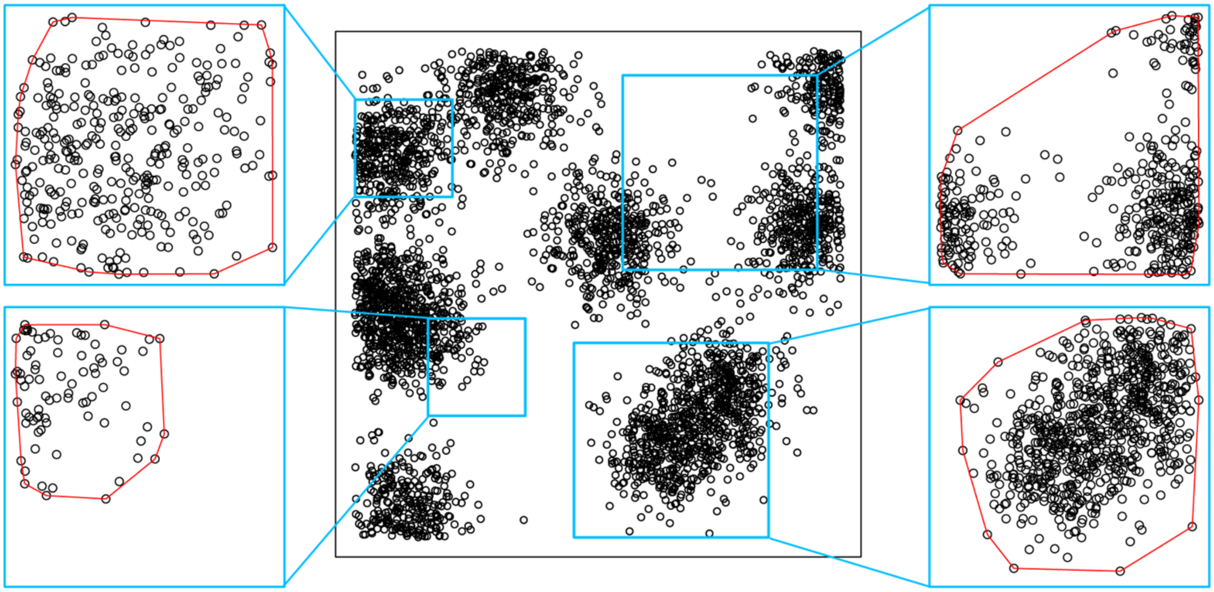

Figure S1.1 Distribution of individuals in a hypothetical population, viewed from a regional scale (middle panel) and a series of sampled landscapes (smaller boxes on left and right). Within each smaller panel, the red line delineates the occupied area that is relevant to isolation-by-distance and effective density estimates.

This scale dependency could matter for measures of isolation-by-distance because IBD is determined by average (effective) population density. A larger study extent that captures greater heterogeneity in population density is likely to have a different average density than a smaller landscape that is continuously populated. This difference is made starker by the fact that the only area that matters for measuring isolation-by-distance is the area between the observed individuals (areas bounded in red in Fig. S1.1). To illustrate, consider the populations in the lower left versus upper right panels of Fig. S1.1. Both landscapes are sparsely occupied, but the lower left panel shows a continuous population, such that the average density of the occupied area remains high, whereas the upper right panel shows a patchy population, such that the average density of the occupied area is lower.

While the landscapes in Fig. S1.1 were chosen specifically to make this point, the pattern described is consistent if landscapes are randomly sampled. In this hypothetical population, sampling a random 1% of the total landscape 1000 times yields an average density of 8312 individuals per unit area, while sampling 81% of the landscape yields an average density of 3472 individuals per unit area (Fig. S1.2). As a result, in this population, IBD would appear stronger (and neighborhood sizes smaller) when estimated across broad spatial extents.

The relationship between spatial extent and average population density (and thus observed IBD) is likely non-linear and in some cases could be non-monotonic. In the example shown here, average density declines rapidly, then levels off or increases slightly (Fig. S1.2). In some iterations of similar simulations, this curve dips or increases at intermediate extents (see section S1.2, below). In real-world populations and landscapes, the effect of spatial extent on IBD estimates will depend on how variance in population density changes with scale (sensu lacunarity; Plotnick et al. 1996). If populations are similarly patchy across a particular range of extents, IBD should not change with extent. Alternatively, if patchiness (and thus sampling variance) does change across a range of extents, so too should observed IBD.

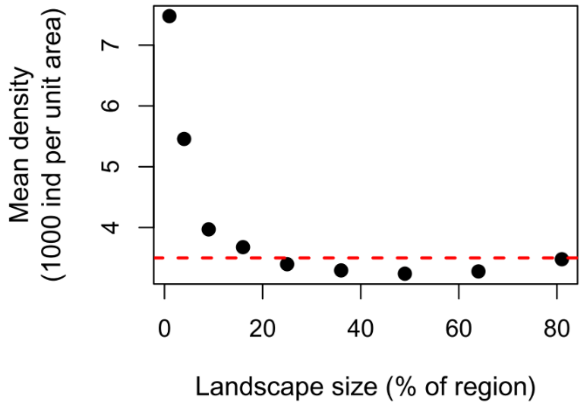

Figure S1.2 As the size of the sampled landscape increases, the more heterogeneity in population density is captured within the focal landscape and so the average population density within the landscape decreases. Values here were taken from 1000 randomly sampled landscapes from the study region in Fig B2. The red dashed line is the true density of the whole occupied region

S1.2 Methods for the simulation

To demonstrate how and why the spatial extent of sampling might affect average population density, and thus rates of isolation by distance, I simulated regional populations and samples of those populations. To simulate the population, I used the sim_thomas_community( ) function in the R package *mobsim* (May et al. 2018, R Core Team 2024), which generates the spatial coordinates for each individual in a simulated community. I included only one species, and so generated coordinates for individuals in a population. In this simulation, no population processes are included, it is simply the spatial distribution. Individuals are distributed patchily, and the level of patchiness is determined by the number of ‘mother points’ around individuals are clustered, and a clustering parameter, sigma. For each simulated population, I included 3500 individuals and 10 mother points.

In S1.1, I show results for one population. To generate this population, I used a clustering parameter (sigma) of 0.065. To test the effects of sampling extent on observed density, I sampled random, square landscapes from the larger region inhabited by the population (i.e., the region is the total area, and the landscape is the sample). I did this 1000 times for each of each of nine

landscape sizes, ranging from 1% of the region to 81% of the region. These percentages come from sampling square landscapes with side length equal to 1/10th to 9/10^{ths} of the total region. Then, to measure density within each sampled landscape, I drew a line around the individuals in the landscape (i.e., a convex hull) to define the occupied area of the landscape, and recorded density as the number of individuals per unit area within the convex hull. I only included a landscape if it contained at least 3 individuals so that I could draw the convex hull and define the occupied area. If a sampled landscape had fewer than 3 individuals, I discarded it and drew a new landscape. As shown in Box 2, I found that the average density was higher in smaller landscapes than in larger landscapes

In addition to what is shown in S1.1, I repeated the above analysis with different levels of population aggregation (i.e., different values of sigma). This was to test for the consistency and generality of the pattern, and the degree to which the effect depends on the strength of population clustering. I found the pattern to be generally consistent, but with more or less concave relationships between scale and density. In a few iterations, seemingly due to idiosyncrasies in the arrangement of habitat clusters, the curves were occasionally non-monotonic, with either a hump or dip at intermediate extents (Fig S1.3).

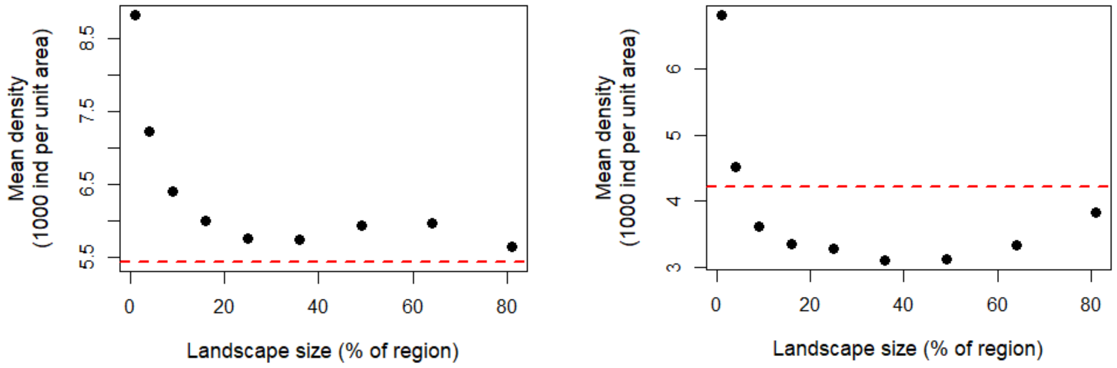

Figure S1.3 Two examples of unusual density-by-landscape size relationships. These specific examples were generated with clustering parameters of sigma = 0.05 (more clustered; left panel) and sigma = 0.08 (less clustered; right panel), but these patterns did not seem to be caused by the degree of clustering specifically. Each of these types of patterns – humps and dips – appeared occasionally in both more and less tightly clustered populations.

S2 Datasets

Table S2.1 Summary of datasets used in this paper. The numbers included reflect the number of specimens from unique colonies that were included in the analysis, not the total number specimens collected. The data themselves are available at https://doi.org/10.5061/dryad.ngf1vhj55, including those data from prior studies for convenience and reproducability. Any use of data from previous studies needs to cite those studies.

Species	Original publication	Data type	Specimens	Sites	Min dist	Max dist
B. bimaculatus	Lozier et al 2011 Molecular Ecology	Microsatellite	448	34	2 km	2201 km
B. impatiens	Lozier et al 2011 Molecular Ecology	Microsatellite	596	34	24 km	2440 km
B. impatiens	This paper	Microsatellite	843	98*	<1 km	146 km
B. occidentalis	Lozier et al 2011 Molecular Ecology	Microsatellite	230	19	16 km	4232 km
B. pensylvanicus	Lozier et al 2011 Molecular Ecology	Microsatellite	304	25	20 km	1759 km
B. vancouverensis	Jackson et al 2018 Molecular Ecology	SNP	382	39	< 1 km	1350 km
B. vosnesenskii	Lozier et al 2011 Molecular Ecology	Microsatellite	319	16	54 km	1505 km
B. vosnesenskii	Jackson et al 2018 Molecular Ecology	SNP	587	50	<1 km	1270 km
B. vosnesenskii	Jha and Kremen 2013 Molecular Ecology	Microsatellite	542	34	<1 km	118 km
B. vosnesenskii	Jha 2015 Molecular Ecology	Microsatellite	754	22	7 km	967 km

* two pairs of siblings were from adjacent sites, so my analyzed dataset included 98 unique locations, rather than 100.

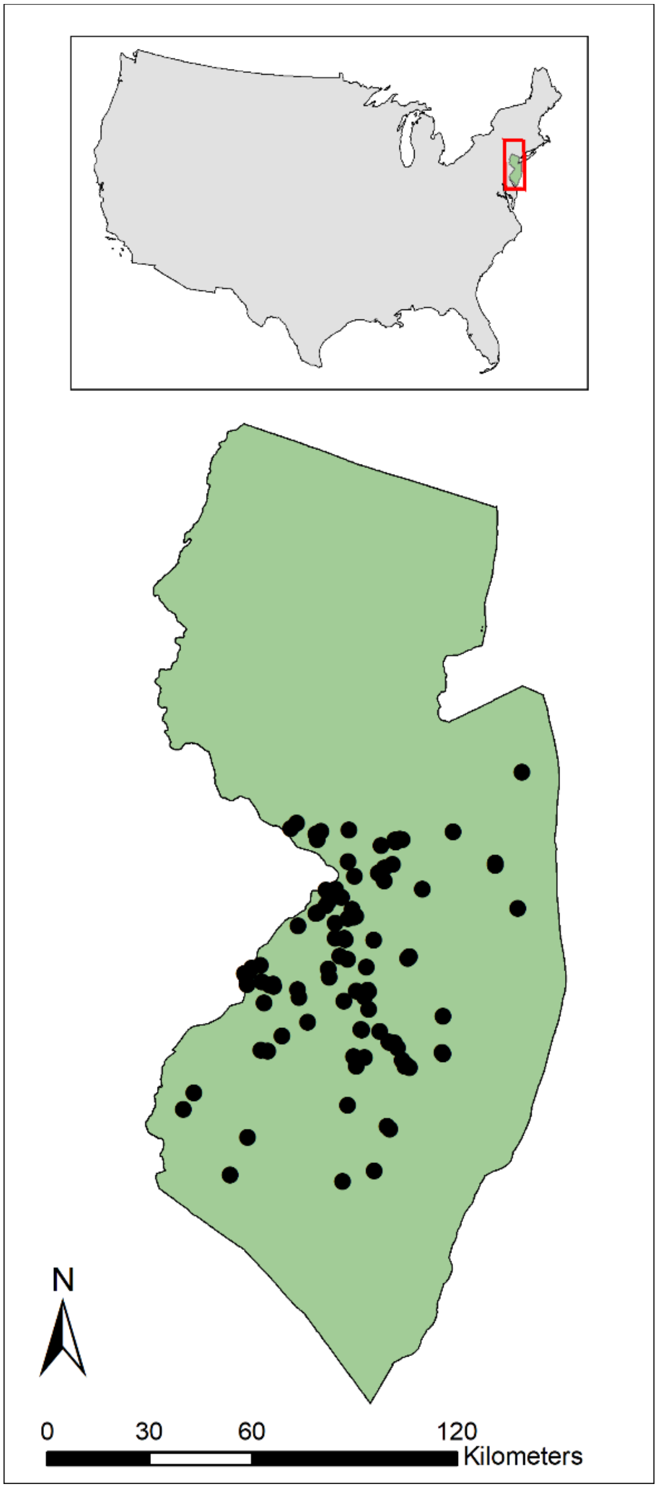

Figure S2.1 The 100 locations at which I collected *Bombus impatiens* in New Jersey

S3 Molecular analysis of New Jersey Bombus impatiens

After collection, each specimen was genotyped at 11 microsatellite loci. I extracted DNA from the two mid legs using an Ampure bead extraction (Ali et al. 2016), then used a multiplex PCR adapted from previous studies (Jha 2015, Mcgrady 2018, McGrady et al. 2021). I used a 20 uL reaction volume, and each reaction included 3 uL DNA extract, 1 U GoTaq Hot Start polymerase, GoTaq Flexi buffer at 1x final concentration, $MgCl_2$ at 1.5 mM, dNTPs each at 300 uM, and each of 22 primers at 0.25 uM. The PCR reaction was as follows: 3 minute 30 second initial denaturation at 95 °C, followed by 30 cycles of 30 seconds at 95 °C, 1 minute 15 seconds at an annealing temperature of 55 °C and 45 seconds at 72 °C, then a final 15-minute extension at 72 °C with a hold temperature at 4 °C. Fragment analysis was conducted by the New Jersey Medical School Genomics Center with an Applied Biosystems SeqStudio-3200. I scored genotypes in the cloud-based Thermo Fisher Connect Microsatellite Analysis software. I defined allele bins manually, then genotypes were assigned automatically but I checked them manually. I also re-genotyped 96 individuals to estimate error rates, which were all < 1%. In the rare cases there was disagreement, I used my judgement to keep whichever seemed more realistic (e.g., I would keep a heterozygote over homozygote genotype). After genotyping, I performed quality assurance using Genepop 4.7.5 (Rousset 2008); I checked each locus for deviation from Hardy-Weinberg equilibrium and the presence of null alleles, and pairs of loci for linkage disequilibrium. None of these tests were significant.

To determine which workers came from the same colony, I assessed siblingship using the program COLONY (Jones and Wang 2010). I ran the program with four independent “long” runs. For each run, I set the program to use the full likelihood calculation, to assume monogamy for males and females, to not allow clones, to use unknown population level allele frequencies, and to use no prior on siblingship probabilities. I also used the setting to “scale down” siblingship size, but this was arbitrary; I tried individual runs with this setting on or off and it did not affect my results. Additionally, I did not allow siblingship among bees collected in different years or separated by > 20 km. Colony assignments agreed between the four runs.

S4 Outside estimates of dispersal

An empirical estimate of σ requires a frequency distribution of observed dispersal distances, and I am aware of only one paper to publish this information (Lepais et al. 2010). These authors followed the genetic mark-recapture method described in the main text introduction; they estimated dispersal of *Bombus pascuorum* queens by first capturing workers in the summer to estimate colony location, then capturing queens in the following spring. By determining siblingship between queens and workers, the queens were assigned to a putative colony location, and dispersal distance was estimated as the distance between the location the queen was captured and the estimated location of the colony. Following this method, Lepais *et al.* estimated the dispersal distance of 177 *B. pascuorum* queens. In their paper, the authors present these as a density histogram, with dispersal distances in 1 km bins of 0 to > 8 km. Using the online tool WebPlotDigitizer (https://apps.automeris.io/wpd/), I extracted the proportional frequency of dispersal within each bin, then multiplied by 177 (their total number of queens for which dispersal was estimated) to convert to absolute frequency.

I used these dispersal distance estimates to generate an independent, empirical estimate of the dispersal parameter σ. To do so, I follow parametric expectations of spatial statistics. While it is tempting to simply fit a dispersal kernel to the observed distance-frequency distribution (i.e. the histogram of dispersal estimates), the definition of σ is actually more nuanced. Assuming isotropic (i.e. radially symmetric) dispersal in two-dimensions, σ^2 is the variance of the dispersal kernel that describes displacement along one dimension (i.e. axial distance) (Rousset 1997, Robledo-Arnuncio and Rousset 2010). This variance is related to, but *not equal to*, the variance in realized Euclidean distance between parent and offspring, that is, the absolute distance between parent and offspring in two dimensions, which what one observes when they estimate dispersal distances in the field (Fig S4.1). This means that observed variance in dispersal distance is not equal to the variance of the dispersal kernel. Instead, these are related such that $2\sigma^2 = var(x) + E(x)^2$, where *x* is Euclidean parent-offspring distance and *E(x)* is expected, or mean, observed parent-offspring distance (Rousset 1997). This means that, given observed dispersal estimates, σ can be estimated as $\hat{\sigma} = \sqrt{\frac{var(x)+E(x)^2}{2}}$.

Following the formula above, I estimated σ of the *B. pascuorum* population described by Lepais *et al* (2010) as $\hat{\sigma} = 2.3$. Due to the limitations of the genetic mark-recapture method, however, this estimate is likely biased low and should be considered a minimum estimate at best. This is also in agreement with Lepais *et al*'s own interpretation, which is that their observations provide a "minimum estimate of maximum dispersal." This is because, in mark recapture-based studies like that of Lepais *et al*, the probability of observing dispersal events tends to decrease with distance. This is in part because longer distance dispersal events are likely to take the focal individual outside the study region, and partly because there are fewer site pairs at longer distances and thus lower effective sampling effort at longer distances. As a result, mark recapture studies will tend to underestimate the frequency of long-distance dispersal. This is not meant to be a criticism of Lepais *et al*'s study, which continues to be an important contribution to the literature. It is instead simply part of the challenge in empirically estimating dispersal.

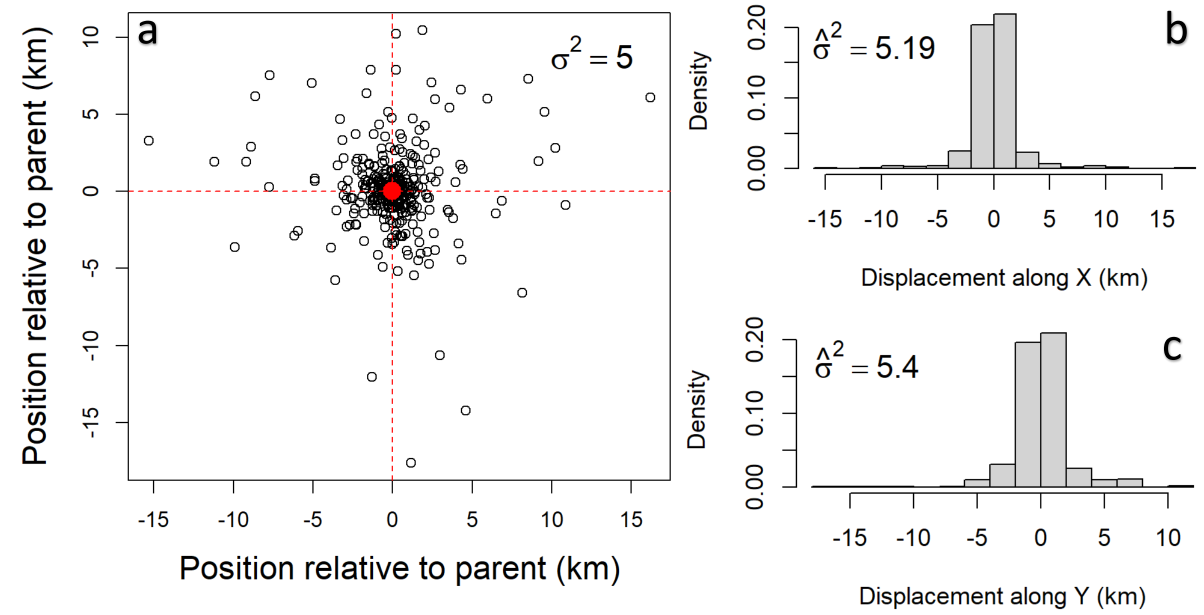

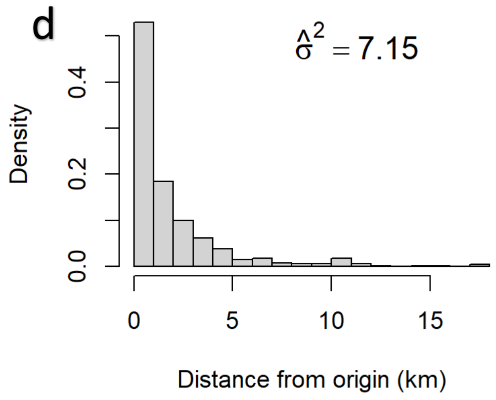

Figure S4.1 Illustration of the relationship between variance of a dispersal kernel, σ^2, and realized Euclidean distance between parent and offspring. (a) Black circles show position of 500 offspring, relative to their parents, following a dispersal kernel with $\sigma^2 = 5$ km. (b-c) Frequency of offspring movement (i.e. axial distance) along two arbitrary axes (red lines in panel a), and their sample variances. (d) Frequency of Euclidean distance between parent and offspring (from panel a) and its sample variance. Because dispersal is observed as Euclidean distance, the underlying σ^2 of the dispersal kernel can be estimated as $\hat{\sigma}^2 = \frac{1}{2}(var(d) + E(d)^2) = 5.3$.

S5 Supplemental Results

TABLE S5.1 Summary of GAMs predicting median effective density (D_e) estimates as a function of the spatial extent of sampling. Models were fit using the gam function in the mgcv package in R 4.4.2. These GAMs are represented in the main text in Figure 2.

Species (dataset)	Effective df	F	p
B. vancouverensis (Jackson)	3.972	979.3	≈ 0
B. impatiens (Lozier)	3.251	26.49	< 0.001
B. occidentalis (Lozier)	3.886	68.72	≈ 0
B. vosnesenskii (Jha 2013)	3.465	18.88	< 0.001
B. vosnesenskii (Jha 2015)	3.238	8.226	0.0013
B. vosnesenskii (Jackson)	1.0	31.04	< 0.001

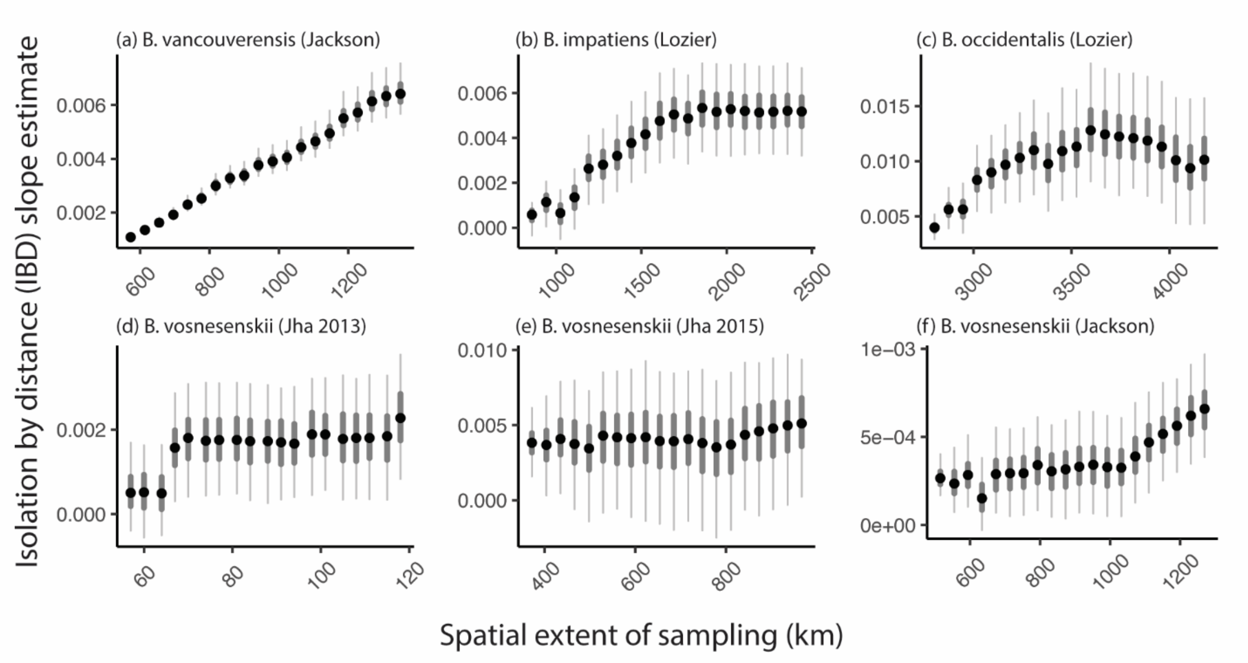

FIGURE S5.1 Observed rates of isolation-by-distance tend to increase with the spatial scale of sampling. In each panel, points show the median estimate across 1000 subsampled pairwise site comparisons at less than or equal to the distance shown on the horizontal axis. Thick grey bars show the inner 50th percentile of estimates at that scale, and thin bars show the inner 95th percentile.

References

Ali, O. A., O'Rourke, S. M., Amish, S. J., Meek, M. H., Luikart, G., Jeffres, C. and Miller, M. R. 2016. Rad capture (Rapture): Flexible and efficient sequence-based genotyping. - Genetics 202: 389–400.

Jha, S. 2015. Contemporary human-altered landscapes and oceanic barriers reduce bumble bee gene flow. - Molecular Ecology 24: 993–1006.

Jones, O. R. and Wang, J. 2010. COLONY: a program for parentage and sibship inference from multilocus genotype data. - Molecular Ecology Resources 10: 551–555.

Lepais, O., Darvill, B., O'Connor, S., Osborne, J. L., Sanderson, R. A., Cussans, J., Goffe, L. and Goulson, D. 2010. Estimation of bumblebee queen dispersal distances using sibship reconstruction method. - Molecular Ecology 19: 819–831.

Levin, S. A. 1992. The Problem of Pattern and Scale in Ecology: The Robert H. MacArthur Award Lecture. - Ecology 73: 1943–1967.

May, F., Gerstner, K., McGlinn, D. J., Xiao, X. and Chase, J. M. 2018. mobsim: An R package for the simulation and measurement of biodiversity across spatial scales (S Goslee, Ed.). - Methods Ecol Evol 9: 1401–1408.

Mcgrady, C. M. 2018. Pollination services, colony abundances, and population genetics of Bombus impatiens.

McGrady, C. M., Strange, J. P., López-Uribe, M. M. and Fleischer, S. J. 2021. Wild bumble bee colony abundance, scaled by field size, predicts pollination services. - Ecosphere in press.

Plotnick, R. E., Gardner, R. H., Hargrove, W. W., Prestegaard, K. and Perlmutter, M. 1996. Lacunarity analysis: A general technique for the analysis of spatial patterns. - Physical Review E 53: 5461–5468.

R Core Team 2024. R: A language and environment for statistical computing.

Robledo-Arnuncio, J. J. and Rousset, F. 2010. Isolation by distance in a continuous population under stochastic demographic fluctuations. - Journal of Evolutionary Biology 23: 53–71.

Rousset, F. 1997. Genetic Differentiation and Estimation of Gene Flow from *F*-Statistics Under Isolation by Distance. - Genetics 145: 1219–1228.

Rousset, F. 2008. genepop'007: a complete re-implementation of the genepop software for Windows and Linux. - Molecular Ecology Resources 8: 103–106.
